# Combined Treatment of 6-Gingerol Analog and Tobramycin for Inhibiting Pseudomonas aeruginosa Infections

**DOI:** 10.1128/Spectrum.00192-21

**Published:** 2021-10-27

**Authors:** So-Young Ham, Han-Shin Kim, Min Jee Jo, Jeong-Hoon Lee, Youngjoo Byun, Gang-Jee Ko, Hee-Deung Park

**Affiliations:** a School of Civil, Environmental and Architectural Engineering, Korea University, Seoul, Republic of Korea; b Korean Peninsula Infrastructure Cooperation Team, Korea Institute of Civil Engineering and Building Technology (KICT), Goyang-si, Gyeonggi-do, Republic of Korea; c Department of Internal Medicine, College of Medicine, Korea University, Seoul, Republic of Korea; d College of Pharmacy, Korea University, Sejong, Republic of Korea; e Biomedical Research Center, Korea University Guro Hospital, Seoul, Republic of Korea; f KU-KIST Graduate School of Converging Science and Technology, Korea University, Seoul, Republic of Korea; University of Maryland School of Pharmacy

**Keywords:** 6-gingerol analog, tobramycin, *Pseudomonas aeruginosa*, biofilm, infection

## Abstract

Pseudomonas aeruginosa is a ubiquitous human pathogen that causes severe infections. Although antibiotics, such as tobramycin, are currently used for infection therapy, their antibacterial activity has resulted in the emergence of multiple antibiotic-resistant bacteria. The 6-gingerol analog, a structural derivative of the main component of ginger, is a quorum sensing (QS) inhibitor. However, it has a lower biofilm inhibitory activity than antibiotics and the possibility to cause toxicity in humans. Therefore, novel and more effective approaches for decreasing dosing concentration and increasing biofilm inhibitory activity are required to alleviate P. aeruginosa infections. In this study, a 6-gingerol analog was combined with tobramycin to treat P. aeruginosa infections. The combined treatment of 6-gingerol analog and tobramycin showed strong inhibitory activities on biofilm formation and the production of QS-related virulence factors of P. aeruginosa compared to single treatments. Furthermore, the combined treatment alleviated the infectivity of P. aeruginosa in an insect model using Tenebrio molitor larvae without inducing any cytotoxic effects in human lung epithelial cells. The 6-gingerol analog showed these inhibitory activities at much lower concentrations when used in combination with tobramycin. Adjuvant effects were observed through increased QS-disrupting processes rather than through antibacterial action. In particular, improved RhlR inactivation by this combination is a possible target for therapeutic development in LasR-independent chronic infections. Therefore, the combined treatment of 6-gingerol analog and tobramycin may be considered an effective method for treating P. aeruginosa infections.

**IMPORTANCE** Pseudomonas aeruginosa is a pathogen that causes various infectious diseases through quorum-sensing regulation. Although antibiotics are mainly used to treat P. aeruginosa infections, they cause the emergence of resistant bacteria in humans. To compensate for the disadvantages of antibiotics and increase their effectiveness, natural products were used in combination with antibiotics in this study. We discovered that combined treatment with 6-gingerol analog from naturally-derived ginger substances and tobramycin resulted in more effective reductions of biofilm formation and virulence factor production in P. aeruginosa than single treatments. Our findings support the notion that when 6-gingerol analog is combined with tobramycin, the effects of the analog can be exerted at much lower concentrations. Furthermore, its improved LasR-independent RhlR inactivation may serve as a key target for therapeutic development in chronic infections. Therefore, the combined treatment of 6-gingerol analog and tobramycin is suggested as a novel alternative for treating P. aeruginosa infections.

## INTRODUCTION

Pseudomonas aeruginosa is an environmental bacterium capable of surviving in any environment, including humans, insects, soil, water, and plants (1). In humans, P. aeruginosa is a major opportunistic pathogen, responsible for 10 to 20% of infections in most hospitals (2). P. aeruginosa is prevalent in patients with burns, immunosuppression, cystic fibrosis (CF), malignancy, and trauma wounds, forming biofilms (3). Biofilm is an adherent bacterial cell aggregation encapsulated in extracellular polymeric substances (EPS) (4, 5). In particular, most CF patients are susceptible to P. aeruginosa biofilms, which have been reported to be associated with over 80% mortality in patients (6). Bacterial biofilms are commonly observed in the lungs of patients with CF (7). Once a P. aeruginosa biofilm is established in the airways of CF patients, complete eradication is difficult. Thus, clinicians generally treat CF patients after the onset of airway biofilm colonization by P. aeruginosa (8).

Antibiotics are generally prescribed to patients to eradicate infection after it emerges (9). Antibiotics kill bacterial cells by inhibiting DNA replication and repair, protein synthesis, and cell wall turnover. Their antibacterial activities contribute to the reduction in biofilm formation (10). In the case of P. aeruginosa-related infections, tobramycin has been routinely used because of its high antibacterial activity. Tobramycin has been reported to be the most active antibiotic against P. aeruginosa isolates from CF patients compared to other antimicrobial agents (e.g., ceftazidime, aztreonam, amikacin, ticarcillin, gentamicin, and ciprofloxacin) (11). Tobramycin has high antibacterial activity, as it leads to a decrease in protein synthesis and disruption of the outer membrane (12). It also completely eradicates the planktonic and biofilm cells of mucoid P. aeruginosa (13). Moreover, Ratjen et al. successfully treated CF patients with P. aeruginosa infection through regular tobramycin inhalation for a year (8). However, poor antibiotic penetration, nutrient limitation, slow growth, adaptive stress responses, and formation of persister cells are known to contribute to decreased antibiotic performance (14). In addition, continuous exposure to antibiotics raises concerns regarding the potential for the emergence of resistant P. aeruginosa (15). Eventually, a decrease in antibiotic prescription is required for long-term patients with P. aeruginosa infections.

To overcome the disadvantages of antibiotics, biofilm inhibitors without bacterial toxic effects have been widely studied over the last 2 decades. Quorum sensing (QS) inhibitors have been introduced as strong antibiofilm agents and as alternatives to antibiotics (16). QS, a bacterial communication system based on signal molecules, is used to coordinate group behaviors to adapt to environmental changes (17). QS is involved in various processes, such as biofilm formation and virulence factor production (18). Therefore, disruption of the QS system results in a significant reduction in biofilm formation and virulence factor production (19). However, QS inhibitors commonly have weaker inhibitory activities on biofilm formation and virulence factor production compared to antibiotics (20). Hence, novel strategies (i.e., combined treatments with QS inhibitors and antibiotics) are required to prevent infections more effectively (21).

**FIG 1:**
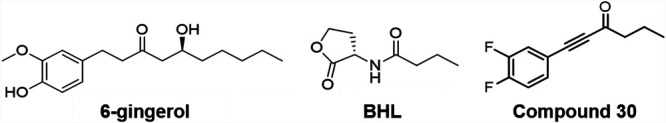
Chemical structures of 6-gingerol, BHL, and compound 30 interacting with the QS receptors of P. aeruginosa.

Our previous study reported that 6-gingerol, the main component of ginger extracts, is a QS inhibitor in P. aeruginosa (22). 6-gingerol consists of a hydroxyl group at the 5-position, a carbonyl group at the 3-position, and a 4′-hydroxy-3′-methoxyphenyl group at the 1-position of its decane backbone structure (Fig. 1). The structure of 6-gingerol is similar to that of QS signal molecules (*N*-oxododecanoyl-l-homoserine lactone (OdDHL) and *N*-butyryl-l-homoserine lactone (BHL)); therefore, it can compete with the QS signal molecules to bind to their cognate receptors. These features of 6-gingerol contribute to the reduction in biofilm formation and production of virulence factors. Furthermore, we developed analogs of 6-gingerol by modifying its structure to facilitate tighter binding of the analogs to QS receptors (23). Compound 30 is a 6-gingerol analog with a difluorine head group, alkynyl ketone, and a short alkyl chain length (Fig. 1) (24). This compound displayed improved binding affinity for RhlR over other QS receptors (LasR and PqsR) with a structure similar to that of BHL (i.e., signal molecule binding to RhlR). Compound 30 strongly inhibited biofilm formation and reduced virulence factor production in P. aeruginosa compared to 6-gingerol. However, prolonged exposure to compound 30 has the potential to cause toxicity in humans. Therefore, the search for methods for treating P. aeruginosa infection sufficiently, even with a low dosing concentration of compound 30, is recommended.

We reasoned that the combination of compound 30 and tobramycin could be successfully applied to treat bacterial infections by controlling biofilm formation and production of virulence factors in P. aeruginosa. In this study, the combined treatment of compound 30 and tobramycin was developed to control P. aeruginosa infection more effectively, and its adjuvant mechanism was investigated. The optimum combination of compound 30 and tobramycin was determined using the fractional inhibitory concentration index (FICI). Adjuvant effects were evaluated by analyzing biofilm formation and the production of virulence factors. Moreover, an adjuvant mechanism was identified through transcriptional analysis. The applicability of the combined treatment was assessed using larval mortality and cytotoxicity tests.

## RESULTS

### Effects of compound 30 and tobramycin on P. aeruginosa.

**FIG 2:**
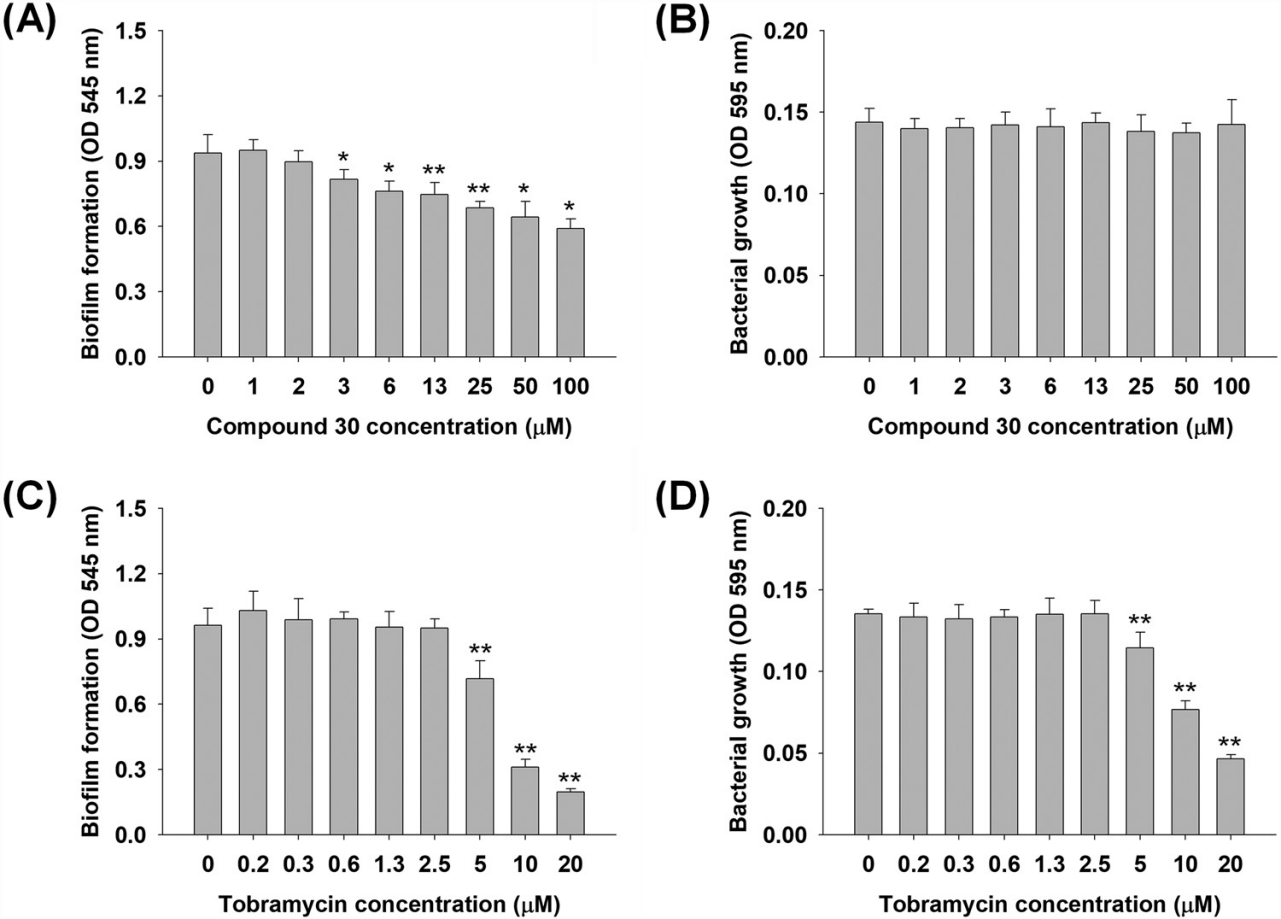
Inhibition of biofilm formation and growth in P. aeruginosa by compound 30 (0 to 100 μM) or tobramycin (0 to 20 μM) for 24 h in static conditions. (A) Biofilm formation by compound 30. The biofilm formation was quantified by measuring the OD at 545 nm of the stained biofilm cells with crystal violet. (B) Bacterial growth by compound 30. Bacterial growth was quantified by measuring the OD at 595 nm of the bacterial suspension. (C) Biofilm formation by tobramycin. (D) Bacterial growth by tobramycin. **, *P* < 0.005; *, *P* < 0.05 compared with the control.

The effects of compound 30 and tobramycin on P. aeruginosa biofilm formation and bacterial growth were investigated by measuring the optical density (OD) at 545 nm and 595 nm, respectively. Compound 30 inhibited biofilm formation at concentrations of >3 μM without affecting bacterial growth (Fig. 2A and B). Biofilm formation decreased in response to the concentration of compound 30. Negligible effects on bacterial growth were observed even at high concentrations (100 μM). In contrast, biofilm formation and bacterial growth started to decrease significantly at tobramycin concentrations of >5 μM (Fig. 2C and D) with similar inhibition patterns, suggesting that tobramycin inhibited biofilm formation through antibacterial activity. Compound 30 significantly reduced P. aeruginosa biofilm formation under static and flow conditions through the disruption of QS processes (24). However, QS inhibitors generally have lower biofilm inhibitory activity than antibiotics (20). The 50% inhibitory concentration (IC_50_) (i.e., half-maximal inhibitory concentration) values of compound 30 and tobramycin against biofilm formation were >100 μM and 7 μM, respectively (Fig. 2A and C).

Tobramycin has been mostly applied to reduce P. aeruginosa biofilm formation based on its high antibacterial activity, although gentamicin and carbenicillin are also used to treat P. aeruginosa infection (25). Gentamicin and carbenicillin decreased biofilm formation and bacterial growth at relatively higher concentrations compared to those of tobramycin (see Fig. S1 in the supplemental material). The minimum inhibitory concentration (MIC) and minimum biofilm eradication concentration (MBEC) values of gentamicin and carbenicillin were 5 to 17 times higher than those of tobramycin in P. aeruginosa (see Table S1 in the supplemental material), confirming that P. aeruginosa is very susceptible to tobramycin among other antibiotics.

### Determination of optimum combination of compound 30 and tobramycin.

Adjuvant combinations of compound 30 and tobramycin (9 conditions of compound 30 × 9 conditions of tobramycin) were evaluated through the FICI equation using their MBEC values (3 μM for compound 30 and 5 μM for tobramycin). As listed in Table 1, three adjuvant combinations (1 μM compound 30 + 0.16 μM tobramycin, 1 μM compound 30 + 0.31 μM tobramycin, and 1 μM compound 30 + 0.63 μM tobramycin) were determined with FICI values of 0.29, 0.32, and 0.39, respectively.

**TABLE 1:** Adjuvant effects between compound 30 and tobramycin

Combination no.	Amount used in combination (μM)		FICI^*a*^	Effect^*b*^
	Compound 30	TOB		
1	1	0.16	0.29	Adjuvant
2	1	0.31	0.32	Adjuvant
3	1	0.63	0.39	Adjuvant

a 3 μM of compound 30 and 5 μM of tobramycin were used to calculate FICI value.

b FICI value < 0.5 was classified as an adjuvant effect.

**FIG 3:**
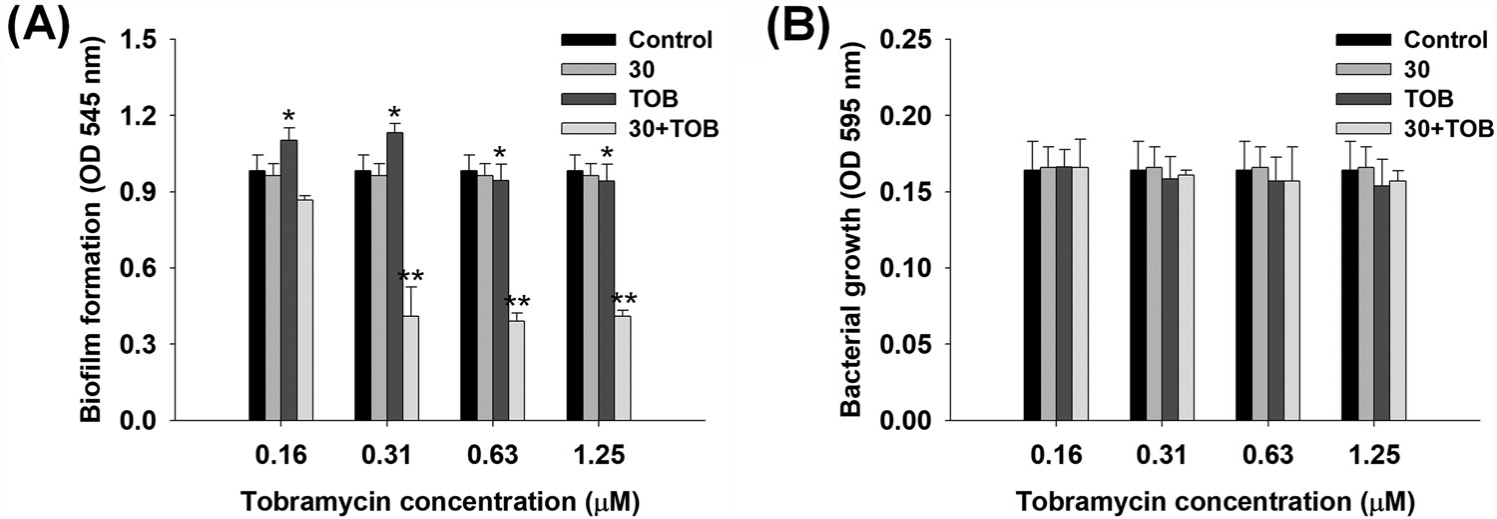
Inhibition of biofilm formation and growth of P. aeruginosa at different combinations of compound 30 (30) (1 μM) and tobramycin (TOB) (0.16, 0.31, 0.63, and 1.25 μM) for 24 h in static conditions. (A) Biofilm formation by the combination of compound 30 and tobramycin. (B) Bacterial growth by the combination of compound 30 and tobramycin. **, *P* < 0.005; *, *P* < 0.05 compared with the control.

A single treatment with compound 30 did not significantly affect biofilm formation, indicating that the concentration of compound 30 (1 μM) was too low to inhibit biofilm formation (Fig. 3A). P. aeruginosa recognizes a low level of antibiotics as toxic compounds and induces biofilm formation to protect itself from environmental pressure (26). As shown in Fig. 3A, upon treatment with 0.16 and 0.31 μM tobramycin, biofilm formation was slightly increased by 12 to 15% compared to that in the control, whereas biofilm formation remained unaffected upon treatment with 0.63 and 1.25 μM tobramycin. Furthermore, treatment with 1 μM compound 30 and 0.63 μM tobramycin resulted in the highest biofilm inhibitory activity (60%) among other combinations with similar biofilm inhibitory activity of the treatment with 1 μM compound 30 and 1.25 μM tobramycin. Bacterial growth was not affected by any combination treatment (Fig. 3B). Therefore, the combination of 1 μM compound 30 and 0.63 μM tobramycin was selected as the optimum adjuvant condition for further studies. In the case of gentamicin and carbenicillin, there were no apparent adjuvant effects when combined with compound 30 (see Fig. S2 and Table S2 in the supplemental material).

### Biofilm inhibition at the optimum combination of compound 30 and tobramycin.

**FIG 4:**
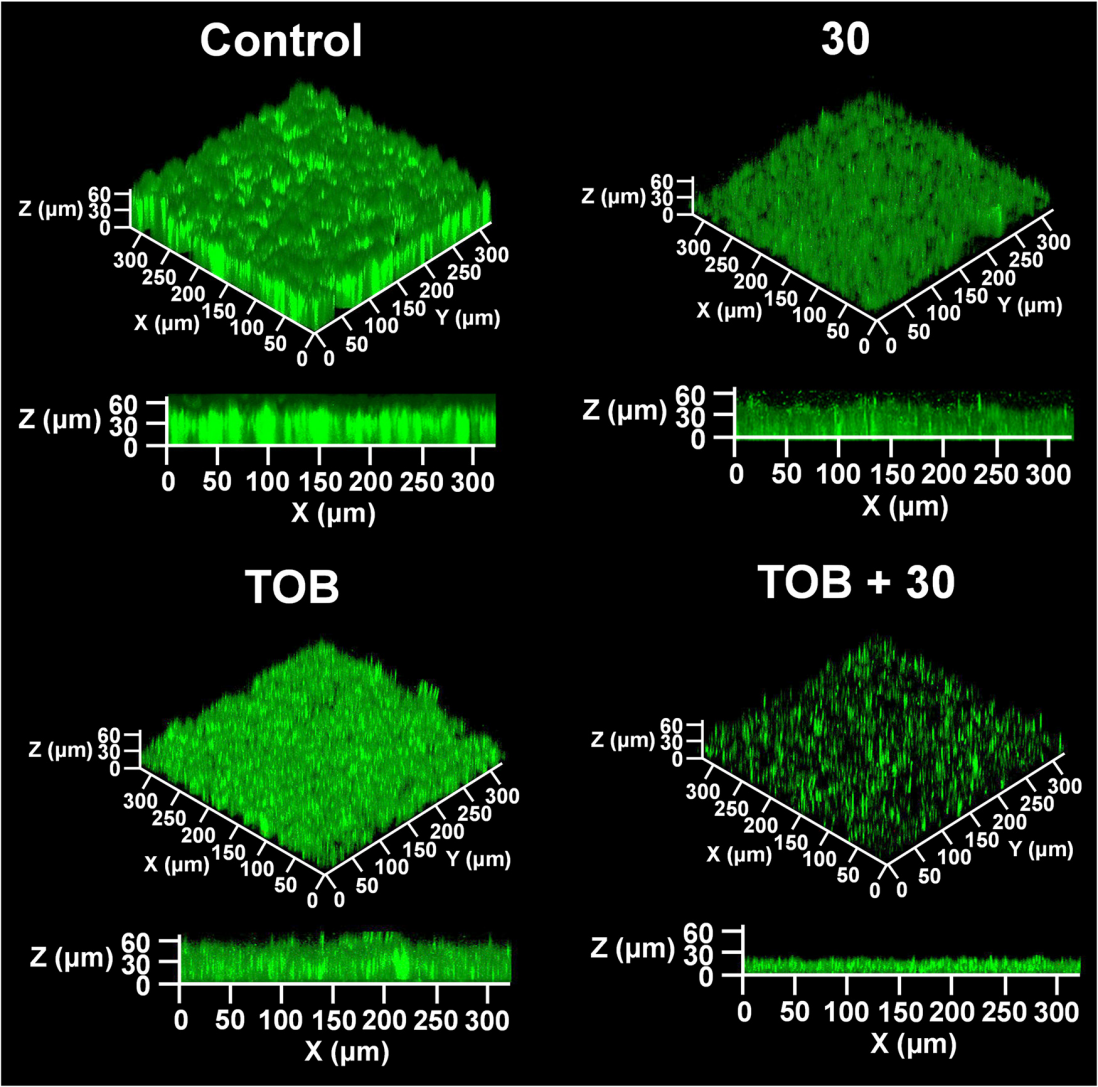
CLSM images of biofilms at the optimum combination of compound 30 (30) (1 μM) and tobramycin (TOB) (0.63 μM) in flow conditions. P. aeruginosa biofilms were formed by operating a drip-flow reactor for 48 h. The biofilm cells were stained with DAPI, and the fluorescent color generated from DAPI was marked in green.

P. aeruginosa biofilm formation under the combination of compound 30 and tobramycin was observed in flow conditions to represent similar conditions of P. aeruginosa infections, such as urinary tract, bloodstream, or catheter biofilm models (27). As shown in Fig. 4, the control biofilm formed mushroom-shaped structures. Biofilm volume and thickness were observed to be slightly inhibited following single treatments (i.e., tobramycin or compound 30 alone) compared to those of the control biofilm (Table 2). However, biofilm treated with the combination were significantly sparser and thinner than any other biofilms in this study. The results of carbohydrate and protein production tests showed patterns similar to those of the confocal laser scanning microscopy (CLSM) images (see Fig. S3 in the supplemental material).

**TABLE 2:** Quantitative analysis of biofilms using ImageJ program

Biofilm treatment	Vol (μm^3^/μm^2^)^*a*^	Thickness (μm)^*a*^
Control	53 ± 2	58 ± 1
Compound 30	27 ± 3**	36 ± 2**
TOB	47 ± 2*	56 ± 0*
Compound 30 + TOB	8 ± 1**	18 ± 2**

a **, *P* < 0.005; *, *P* < 0.05 as compared with the control.

The carbohydrate and protein contents of biofilms treated with compound 30 or tobramycin decreased by 15 to 24% and 12 to 26% compared to those in the control, respectively, while biofilm treated with the combination showed high inhibition of carbohydrate and protein contents (50 to 52%). As shown in Table 2, biofilm volume and thickness of biofilms cells decreased concordant to the results of EPS analysis (Fig. S3). Moreover, there were no significant changes in bacterial growth and colony cell numbers in both planktonic and biofilm cells (Fig. 3B; see also Fig. S4 in the supplemental material) when treated with the optimum combination, suggesting that the combination of compound 30 and tobramycin may be adjuvanted via other mechanisms without increasing antibacterial activity.

### QS inhibition at an optimum combination of compound 30 and tobramycin.

**FIG 5:**
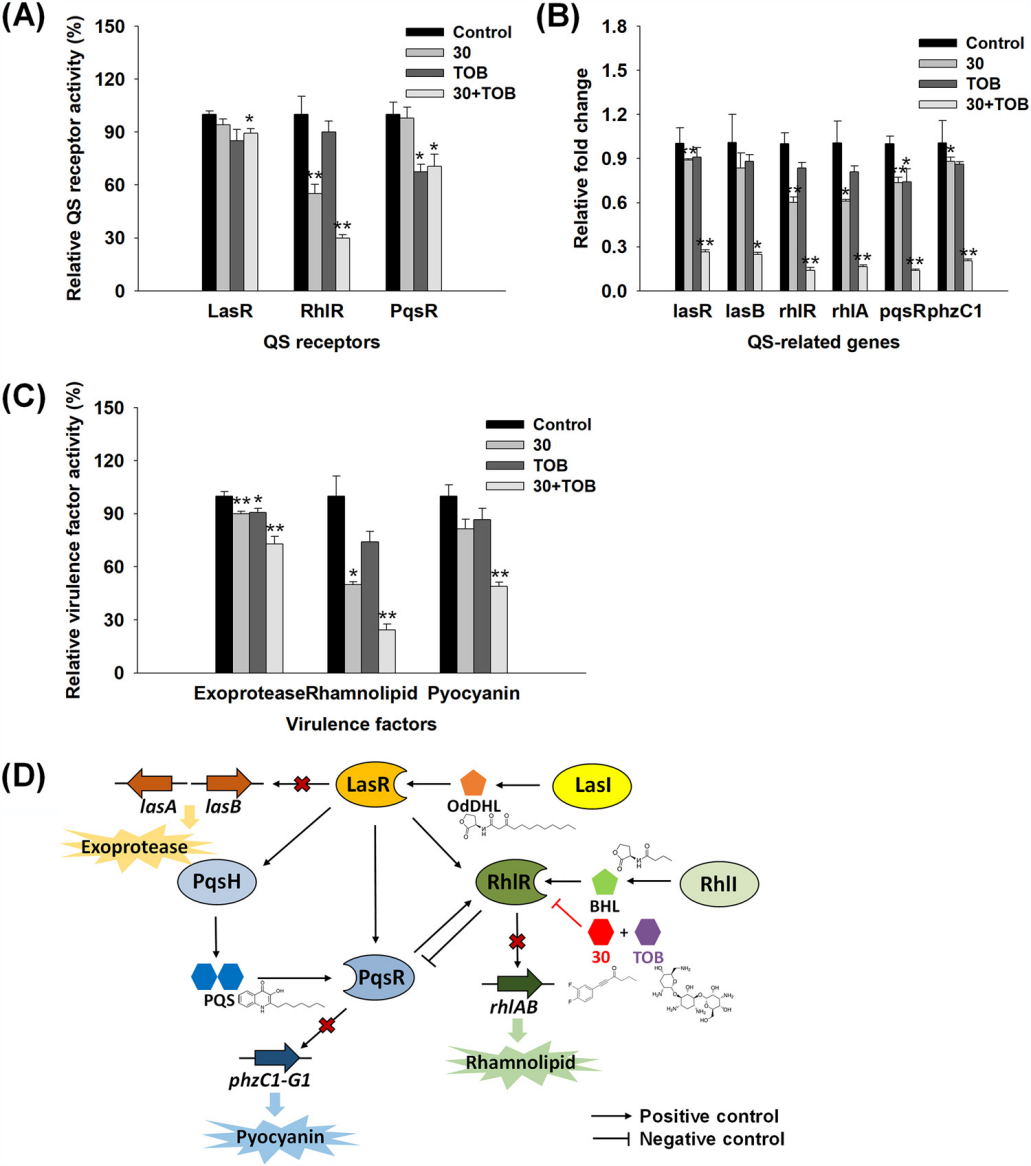
Adjuvant effects in P. aeruginosa at the optimum combination of compound 30 (30) (1 μM) and tobramycin (TOB) (0.63 μM). (A) Antagonism activities against the QS receptors, LasR, RhlR, and PqsR using OdDHL, BHL, and PQS as their respective signal molecules. (B) QS-related gene expression levels of P. aeruginosa biofilm cells subjected to combined treatments. Biofilm cells were formed in borosilicate bottles for 24 h, and gene expression was analyzed using RT-qPCR. (C) Virulence factor production (exoprotease, rhamnolipid, and pyocyanin) in P. aeruginosa biofilm cells. Exoprotease and rhamnolipid were estimated by measuring the color changes of the reactant with azocasein and ordinal solution, respectively. Pyocyanin was estimated through HPLC analysis. **, *P* < 0.005; *, *P* < 0.05 compared with the control. (D) Schematic diagram of the alternation of QS gene expression and virulence production by the adjuvant combination of compound 30 and tobramycin.

QS receptor binding test and reverse transcriptase quantitative PCR (RT-qPCR) were conducted at the optimum combination of compound 30 (1 μM) and tobramycin (6.3 μM). Figure 5A shows the binding activities of the chemicals toward representative QS receptors (LasR, RhlR, and PqsR). Compound 30 displayed selective RhlR antagonism over LasR and PqsR in P. aeruginosa, which is consistent with the results of our previous study (24). Following combined treatment with compound 30 and tobramycin, antagonistic binding activities were observed in LasR, RhlR, and PqsR. In particular, high antagonism activity was measured in RhlR binding, indicating that tobramycin has positive effects on the RhlR binding affinity of compound 30. In contrast, there were no agonism-binding activities against any of the QS receptors (see Fig. S5 in the supplemental material).

RT-qPCR analysis showed that QS-related gene expression was significantly downregulated in the combined treatment (74 to 86%) compared to that in the single treatment (12 to 63%) (Fig. 5B). In particular, decreasing trends clearly appeared in the expression of *rhl*- and *pqs*-associated genes (*rhlR*, *rhlA*, *pqsR*, and *phzC1*). P. aeruginosa produces many virulence factors (e.g., exoprotease, rhamnolipid, and pyocyanin), which are controlled by QS systems (28). The production of virulence factors decreased by 13 to 50% in single treatments, whereas it was decreased by 27 to 76% in the combined treatment as shown in Fig. 5C. These results suggested that the adjuvant effects of compound 30 and tobramycin were generated via the interruption of QS systems. Cyclic-di-GMP (c-di-GMP) is a secondary messenger signaling system that regulates biofilm formation in P. aeruginosa (29). Although c-di-GMP levels in P. aeruginosa with combined treatment slightly decreased by only 10 to 20% compared to the control, the degree of decline was not significant (see Fig. S6 in the supplemental material).

### Application of combined treatment of compound 30 and tobramycin on P. aeruginosa infections.

**FIG 6:**
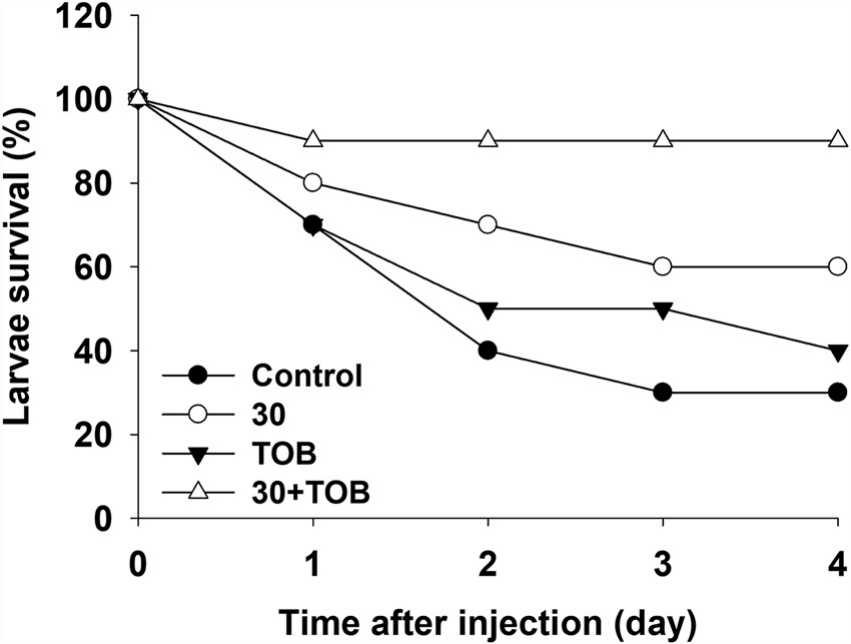
The survival rate of *T. molitor* larvae injected with P. aeruginosa at the optimum combination of compound 30 (30) (1 μM) and tobramycin (TOB) (0.63 μM). The larval survival rate was estimated by counting the numbers of melanized larvae for 4 days.

The combination of compound 30 and tobramycin was applied to Tenebrio molitor larvae infected with P. aeruginosa. Larvae began to die from the beginning, following the injection of P. aeruginosa, and 70% of them died after 4 days (Fig. 6). The survival rate of compound 30- or tobramycin-treated larvae was 40 to 60%. However, when treated with the combination of compound 30 and tobramycin, the survival rate of larvae improved significantly. At the end of the study period, 90% of the larvae survived.

**FIG 7:**
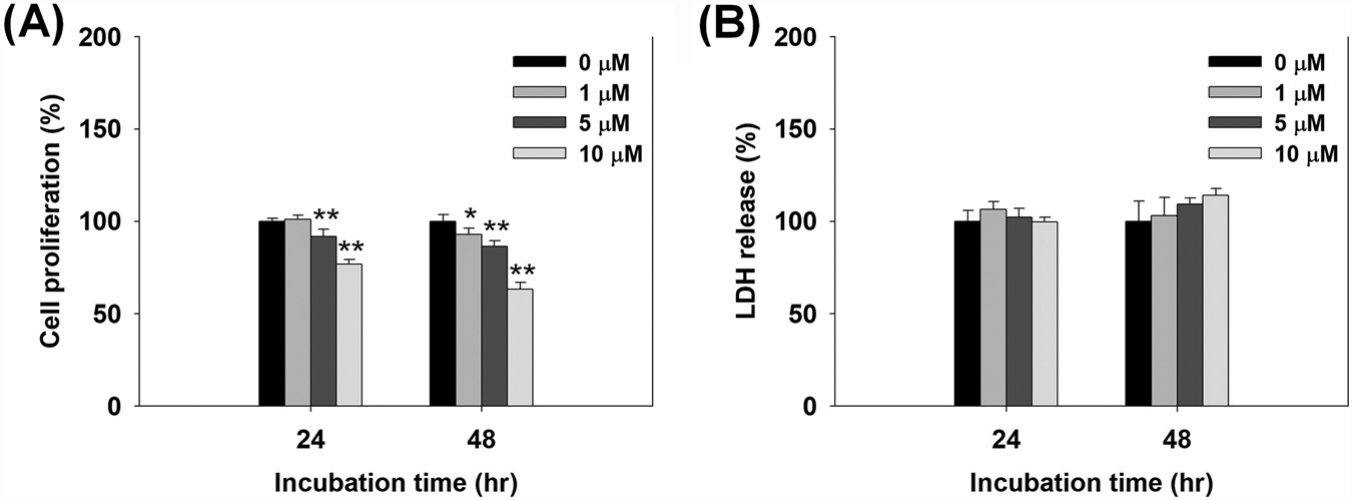
Cytotoxic effects of compound 30 (0 to 10 μM) on human lung epithelial cells. Lung cells were incubated with compound 30 for 24 and 48 h in microtiter plates, and their color changes were measured. (A) The proliferation of lung cells treated with compound 30 by MTS assay. (B) LDH release of lung cells treated with compound 30 by LDH cytotoxicity assay. **, *P* < 0.005; *, *P* < 0.05 compared with the control.

The cytotoxicity of compound 30 against human lung epithelial cells was assessed using the 3-(4,5-dimethylthiazol-2-yl)-5-(3-carboxymethoxyphenyl)-2-(4-sulfophenyl)-2H-tetrazolium, inner salt (MTS) cell proliferation assay and by measuring lactate dehydrogenase (LDH), a cytosolic enzyme released from damaged or destroyed cells due to cell membrane destruction (30). Although the proliferation of cells treated with 10 μM compound 30 (i.e., 10 times higher concentration than that in adjuvant condition) was inhibited by 23 to 37%, there was no significant decrease in the proliferation of cells treated with 1 and 5 μM compound 30 (Fig. 7A). LDH release was also constant in cells treated with 1 and 5 μM compound 30 compared to that in the control after 24 and 48 h of incubation, although LDH release in cells treated with 10 μM compound 30 was slightly increased by 14% at 48 h (Fig. 7B). Furthermore, combined treatment with compound 30 and tobramycin slightly increased cell proliferation, and LDH release was constant at both 24 and 48 h of incubation (see Fig. S7 in the supplemental material), suggesting that the combined treatment had no cytotoxic effects on lung epithelial cells. These results indicate the possibility of safe application of compound 30 for the treatment of CF patients, expecting that compound 30 is considered tolerable for cytotoxicity under adjuvant conditions. However, the continuous administration of compound 30 to patients can cause concerns about toxicity in humans. Therefore, careful monitoring of cytotoxicity by compound 30 is required for its application in CF patients with P. aeruginosa infections.

## DISCUSSION

This study clearly demonstrated that compound 30 showed adjuvant effects in decreasing biofilm formation and virulence factor production in P. aeruginosa when combined with tobramycin. Tobramycin is a commonly used antibiotic for treating P. aeruginosa infections because of its high antibacterial activity (25). Although tobramycin is currently used in CF lung infections, it is difficult to completely heal P. aeruginosa infections (31). Thus, combined treatments with QS inhibitors have been shown to be effective in attenuating biofilm formation (32, 33) because the interruption of QS also plays an important role in the treatment of CF patients with P. aeruginosa infection (34). The adjuvant effects of QS inhibitors and antibiotics have been studied by many researchers. Brackman et al. identified that combined treatment with QS inhibitors and antibiotics results in increased killing effects compared to treatment with antibiotics alone (20). Christensen et al. discovered the adjuvant effects of furanone C-30 on the retardation of P. aeruginosa biofilm formation when used in combination with tobramycin (35). Early initiation of treatment with the combination of furanone C-30 and tobramycin improved the immune response in a foreign-body infection mouse model compared to that with single treatments. They generally focused on the adjuvant mechanism that involved QS inhibition, which in turn induces the success of antibiotic treatment due to the increased susceptibility of bacterial biofilms. However, our results showed that adjuvant effects occurred, although there was little effect on the antibacterial activity of the antibiotics (Fig. 3).

Compound 30, an RhlR antagonist of P. aeruginosa, inhibits biofilm formation and virulence factor production via the disruption of the QS system (24). Based on the biofilm inhibition mechanism of compound 30, it can be hypothesized that adjuvant effects are related to QS systems. When treated with compound 30 and tobramycin, RhlR binding affinity was greatly improved compared to single treatments, indicating that tobramycin appears to have positive effects on the binding of compound 30 to RhlR (Fig. 5A). Furthermore, the biofilm inhibition activity of tobramycin-resistant P. aeruginosa strains by compound 30 was similar to that of wild-type P. aeruginosa, but adjuvant biofilm inhibitory activity was decreased by 19 to 38% in tobramycin-resistant P. aeruginosa strains (36) (see Fig. S8 in the supplemental material). These results also suggest that tobramycin can potentially facilitate the activity of compound 30. Tobramycin has been reported to alter the permeability of the outer membrane of P. aeruginosa (37–39). Outer and inner membrane permeability in P. aeruginosa increased in proportion to the concentration of tobramycin, while there were no changes even after treatment with 100 μM compound 30 (see Fig. S9 in the supplemental material). Therefore, an increase in permeability by tobramycin may enhance the transport of compound 30 into the cytoplasm, contributing to easier access and binding of compound 30 to RhlR.

Several researchers have focused on the adjuvant effects of QS inhibitors and antibiotics based on QS systems. Yang and coworkers discovered analogs of signal molecules as novel QS inhibitors (40). They identified that adjuvant interactions between analogs and antibiotics successfully reduced the production of virulence factors in P. aeruginosa by controlling the QS system. Chanda et al. revealed that linoleic acid, a natural fatty acid from ginger extracts, improved the potency of tobramycin when added together in P. aeruginosa biofilms (41). They confirmed that combined treatment downregulated QS system-associated gene expression more effectively. Furthermore, Topa et al. proposed a combination therapy of cinnamaldehyde with tobramycin as an alternative to mitigate P. aeruginosa infections (42). Tobramycin showed strong QS inhibitory activity when combined with cinnamaldehyde. Similar to previous studies, the combination of compound 30 and tobramycin leads to adjuvant effects via interruption of the QS system, eventually decreasing QS-related gene expression and production of virulence factors (Fig. 5D).

However, once P. aeruginosa colonizes CF airways, it appropriately changes its gene expression to adapt to the environmental conditions of CF patients. Eventually, P. aeruginosa with acute infections shifts its life to suit chronic infections. LasR, the top hierarchy of QS receptors, is mutated in chronic infections, suggesting that QS does not seem to be important in chronic infections (43). In fact, *lasR* mutations are frequently observed in CF patients with chronic infections, whereas *rhlR* mutations are rare. Cabrol et al. reported that only about 50% of clinical isolates were related to *lasR*-independent *lasAB* expression, indicating that *lasR* is no longer an important factor causing infection (44). Alternatively, RhlR, another receptor in QS systems, controls pathology and biofilm formation in P. aeruginosa in an LasR-independent manner (45). Furthermore, RhlR plays an important role in regulating the cytotoxicity of lung epithelial cells (46). Compound 30, a strong RhlR antagonist, effectively reduced biofilm formation and production of virulence factors in P. aeruginosa (24), indicating that compound 30 may be used as a possible target for therapeutic development in chronic infections. Furthermore, when compound 30 was treated with tobramycin, biofilm formation and production of virulence factors greatly decreased compared to those in single treatments, through improved RhlR antagonism activity (Fig. 5). Therefore, if compound 30 is prescribed with tobramycin to CF patients, P. aeruginosa infection might be effectively treated without the adverse effects of antibiotics.

Compound 30 is a QS inhibitor that is synthesized to develop potent antibiofilm agents based on the structure of 6-gingerol. Tobramycin is a representative antibiotic used to prevent P. aeruginosa infections. Combined treatment with compound 30 and tobramycin greatly attenuated biofilm formation and virulence factor production in P. aeruginosa compared to single treatments. Furthermore, it increased the survival rate of *T. molitor* larvae 3-fold without affecting the cytotoxicity of human lung epithelial cells. Strong adjuvant activities of the combination were detected upon disrupting the QS process, although it did not increase the antibacterial activity. Therefore, the combination of compound 30 and tobramycin has the potential to be utilized as a novel antivirulence strategy to combat P. aeruginosa infections.

## MATERIALS AND METHODS

### Bacteria and chemicals.

P. aeruginosa PA14 is a representative bacterium that forms biofilms via QS systems (47). The PA14 strain was incubated in AB medium (300 mM NaCl, 50 mM MgSO_4_, 0.2% casamino acids, 10 mM potassium phosphate, 1 mM l-arginine, and 1% glucose, pH 7.5) at 37°C for 12 h under constant shaking (200 rpm) (48).

Compound 30, a 6-gingerol analog, was synthesized to improve RhlR receptor binding affinity (24) and was dissolved in dimethyl sulfoxide (DMSO) (Carl Roth, Karlsruhe, Germany). Tobramycin (Sigma-Aldrich, St. Louis, MO, USA) was dissolved in deionized water (DW).

### Antibacterial and antibiofilm tests.

To measure the MIC and MBEC of compound 30 or tobramycin, a broth microdilution method was used according to the guidelines of the Clinical and Laboratory Standards Institute (CLSI) M27-A3 (49). One hundred microliters of AB medium was added to each well in a 96-well plate (Sigma-Aldrich). Compound 30 (0 to 100 μM) or tobramycin (0 to 20 μM) was serially diluted 2-fold from left to right in the wells. Overnight cultured PA14 strain was diluted with AB medium to adjust the optical density (OD) at 595 nm to a value of 0.001 (∼10^6^ CFU/ml). One hundred microliters of diluted PA14 strain was added to the plate to achieve a final bacterial concentration of ∼5 × 10^5^ CFU/ml, which was incubated at 37°C for 24 h without agitation (50). MIC was obtained by estimating the OD of the bacterial suspension at 595 nm using a Victor ×5 multimode plate reader (PerkinElmer, Waltham, MA, USA) and then counting the colonies of bacterial cells on the surface of agar plates. MBEC was defined as OD at 545 nm after washing attached biofilm cells with phosphate-buffered saline (PBS) (137 mM NaCl, 2.7 mM KCl, 10 mM Na_2_HPO_4_, and 2 mM KH_2_PO_4_, pH 7.2), staining the biofilm cells with 0.1% crystal violet for 10 min, washing stained biofilm cells with DW, and diluting crystal violet with 100% ethyl alcohol. IC_50_ (concentration causing 50% reduction compared with the control) was calculated from the graph of the dose-response curve generated from biofilm formation against the concentrations of compound 30 or tobramycin.

### Determination of fractional inhibitory concentration index.

The adjuvant effects of compound 30 and tobramycin were evaluated based on the results of the antibiofilm test. The adjuvant interactions were assessed as FICI using the following equation:

FICI = FIC (compound 30) + FIC (tobramycin)

where FIC (compound 30) is the MBEC of compound 30 in combination/MBEC of compound 30 alone, and FIC (tobramycin) is the MBEC of tobramycin in combination/MBEC of tobramycin alone. The optimum adjuvant conditions were determined based on FICI as follows: adjuvant effect (FICI < 0.5), indifferent effect (0.5 ≤ FICI ≤ 4), and antagonistic effect (FICI > 4).

### Dynamic biofilm formation test.

Biofilm formation patterns under flow conditions were observed by operating a drip-flow reactor (DFR-110; BioSurface Technologies, Bozeman, MT, USA) using glass slides. Overnight cultured PA14 strain (OD at 595 nm = 0.05) diluted in AB medium with either compound 30 (1 μM) or tobramycin (0.63 μM) treatments (no treatment, single treatments of compound 30 or tobramycin, and combined treatment of compound 30 and tobramycin) was continuously fed into the reactor using a peristaltic pump (Masterflex C/L tubing pumps; Cole-Parmer, Vernon Hills, IL, USA) at 0.3 ml/min, and the reactor was operated at 37°C for 48 h. The biofilm cells formed on the slides were washed with PBS and stained with 2 μg/ml 4′,6-diamidino-2-phenylindole (DAPI) solution (Carl Roth) for 10 min. After washing the biofilm cells with DW, the stained cells were observed via confocal laser scanning microscopy (CLSM) (Carl Zeiss LSM700, Jena, Germany) using a 20× objective lens (W N-Achroplan × 20/0.5W [DIC] M27) under blue fluorescence light (excitation wavelength, 350 nm; emission wavelength, 470 nm). Biofilm volume and thickness were analyzed using Zen 2011 software (Carl Zeiss) and quantified using Comstat2 in the ImageJ program (51) based on the CLSM images.

### QS receptor binding test.

Binding tests between chemical (compound 30 or tobramycin) and QS receptor (LasR, RhlR, or PqsR) were conducted using three specific reporter strains. Escherichia coli DH5α cotransformed with pJN105L (LasR expression plasmid) and pSC11 (*lasI*::*lacZ* fusion plasmid), E. coli DH5α *sdiA* mutant with pJN105R (RhlR expression plasmid) and pECP60 (*rhlA*::*lacZ* fusion plasmid), and E. coli DH5α with pJN105P (PqsR expression plasmid) and pJN301 (*pqsA*::*lacZ* fusion plasmid) were used as the LasR, RhlR, and PqsR reporter strains, respectively (52–54). For the antagonism test, the chemicals (1 μM compound 30 or 0.63 μM tobramycin) and the signal molecule (0.01 μM OdDHL for LasR, 10 μM BHL for RhlR, or 1 μM pseudomonas quinolone signal [PQS] for PqsR) (Sigma-Aldrich) were treated with the reporter strain (OD at 595 nm = 0.3). Meanwhile, the reporter strain was treated with chemicals (1 μM compound 30 or 0.63 μM tobramycin) alone, and the signal molecule was used as a positive control in the agonism test. The reacted strain was added to 0.4% arabinose (Sigma-Aldrich) and incubated at 37°C for 1.5 h. The OD at 595 nm of the incubated reporter strain (100 μl) was measured using a spectrophotometer (Shimadzu, Kyoto, Japan). The remaining reporter strain was reacted with 10 μl chloroform (Sigma-Aldrich) for 10 min and vortexed for 10 s. Ten microliters of the suspension was used to measure luminescence using a Tropix Galacto-Light luminescent assay kit (Applied Biosystems, CA, USA). Relative QS receptor activity was quantified as luminescence normalized to the OD at 595 nm.

### RT-qPCR.

An overnight culture of the PA14 strain (OD at 595 nm = 0.05) diluted in AB medium with either compound 30 or tobramycin treatment was incubated at 37°C for 24 h without agitation in borosilicate bottles. After washing the biofilm cells with PBS, the cells attached to the bottles were scraped and collected in a microtube. Total RNA was extracted from the biofilm cells by using TRI reagent (Molecular Research Center, OH, USA) following the manufacturer’s instructions. The reaction mixture consisted of 10 μl of SYBR Premix Ex Taq (TaKaRa, Shiga, Japan), 1 μl of forward and reverse primers (10 μM), 2 μl of extracted RNA (100 ng/μl), and RNase-free water to generate a final volume of 20 μl. QS-related gene primer sequences were identical to those used in our previous study (see Table S3 in the supplemental material) (22). Thermal profiles of RT-qPCR were as follows: initial denaturation at 95°C for 10 s, denaturation at 95°C for 10 s, annealing at 60°C for 10 s, and extension at 63°C for 34 s. Fluorescence signal intensity was measured at the end of the extension step using a CFX-96 real-time system (Bio-Rad, Hercules, CA, USA) and normalized with *proC* as a housekeeping gene (55).

### Analysis of the production of virulence factors.

An overnight cultured PA14 strain (OD at 595 nm = 0.01) diluted in AB medium under different treatment conditions was incubated at 37°C for 24 h under constant shaking (200 rpm). The OD of the suspended cells at 595 nm was measured using a spectrophotometer. Following centrifugation at 4°C for 10 min, the supernatant was filtered through a 0.22-μm membrane filter. Filtered supernatants were used for virulence factor production tests (exoprotease, rhamnolipid, and pyocyanin).

For the exoprotease production test, 250 μl of the supernatant with 250 μl of 0.5% (wt/vol) azocasein (Sigma-Aldrich) in 50 mM Tris-HCl (pH 8.0) was incubated at 37°C for 2 h. After adding 500 μl of 10% trichloroacetic acid (Sigma-Aldrich) solution and incubating on ice for 30 min, the mixture was centrifuged at 10,000 rpm for 10 min. The supernatant (500 μl) was mixed with 500 μl of 1 M NaOH. Exoprotease was quantified as OD at 440 nm and normalized to the OD at 595 nm.

For the rhamnolipid production test, rhamnolipids were extracted using 500 μl of supernatant and 1,000 μl of 100% diethyl ether (Sigma-Aldrich). The dried ether fraction in the upper layer was mixed with 150 μl of DW and 1,350 μl of 0.19% ordinal solution (Sigma-Aldrich) dissolved in 53% H_2_SO_4_. The mixture was boiled at 80°C for 30 min and then cooled at 25°C for 15 min. Rhamnolipid was quantified as OD at 421 nm and normalized to OD at 595 nm.

For the pyocyanin production test, 5 ml of the supernatant was reacted with 30 μl of a 50% trifluoroacetic acid (TFA) (Sigma-Aldrich) solution at 25°C for 1 h. The supernatant was centrifuged for 10 min at 4,750 rpm and then passed through a 0.22-μm membrane filter. A 1260 Infinity II Prep-HPLC system (Agilent Technologies, Santa Clara, CA, USA) and an EC-C_18_ column (4.6 × 150 mm; Agilent Technologies) were used to analyze the filtered samples. The detailed high-pressure liquid chromatography (HPLC) conditions were as follows: 99:1 water/TFA (vol/vol) mobile phase, 10 μl injection volume, 25°C temperature, and 0.5 ml/min flow rate. The pyocyanin peak at a retention time of 20 min was detected with a UV-visible (UV-vis) detector, and the height of the peak was estimated for pyocyanin measurement.

### *T. molitor* larvae survival test.

PA14 strain (OD at 595 nm = 0.01) cultured overnight was diluted in AB medium with either compound 30 or tobramycin treatment and incubated at 37°C for 24 h under constant shaking (200 rpm). The incubated PA14 strain (OD at 595 nm = 1.0) was centrifuged for 10 min at 8,000 rpm, and the supernatant was passed through a 0.22-μm membrane filter. Ten microliters of the filtered supernatant was injected into *T. molitor* larvae (1 to 1.5 cm length) using a microsyringe needle. Larvae were maintained in a terrarium containing wheat bran on a laboratory bench. After injection, the larvae were incubated in a petri dish at 25°C for 5 days. The survival rate of the larvae was determined by counting the number of melanized larvae every day.

### Cell cytotoxicity test.

The cytotoxicity of compound 30 in human lung epithelial cells (BEAS-2B; American Type Culture Collection [ATCC], Manassas, VA, USA) was assessed using MTS cell proliferation, and LDH release tests. The MTS assay was performed to determine cell viability using a Cell Titer 96 aqueous one-solution cell proliferation assay kit (G3580; Promega, Madison, WI, USA). Human lung epithelial cells (3 × 10^3^ cells/well) were seeded into a 96-well plate and treated with 1, 5, or 10 μM compound 30. Following incubation at 37°C for 24 and 48 h, cells were stained with 20 μl of MTS for 1 h. Cell proliferation was evaluated by measuring the OD at 490 nm using a microplate reader (SpectraMax 190; Molecular Devices, CA, USA).

The LDH release test was performed to determine cellular cytotoxicity using the CyQuant LDH cytotoxicity assay kit (C20300; Invitrogen, Carlsbad, CA, USA). Human lung epithelial cells treated with compound 30 (1, 5, or 10 μM) were seeded into a 96-well plate and lysed in lysis buffer. After incubation at 37°C for 45 min, 50 μl of the reaction mixture was added to each well. The reaction was stopped using a stop solution. LDH release was evaluated by measuring the OD at 490 nm with an OD of 680 nm as the reference wavelength.
