# Supplementary material for: Combined Treatment of 6-Gingerol Analog and Tobramycin for Inhibiting Pseudomonas aeruginosa Infections
Source: Microbiol Spectr. 2021 Oct 27;9(2):e00192-21. doi: 10.1128/Spectrum.00192-21 (PMC8549756; doi:10.1128/Spectrum.00192-21)
Supplement: SUPPLEMENTAL FILE 1 — Supplemental material. Download Spectrum.00192-21-s0001.pdf, PDF file, 0.3 MB [file spectrum.00192-21-s0001.pdf]

Supporting Information

Combined treatment of 6-gingerol analog and tobramycin for inhibiting *Pseudomonas aeruginosa* infections

So-Young Ham[a], Han-Shin Kim[b], Min Jee Jo[c], Jeong-Hoon Lee[a], Youngjoo Byun[d,e]
Gang-Jee Ko[c,*], and Hee-DeungPark[a,f,*]

[a]School of Civil, Environmental and Architectural Engineering, Korea University, 145 Anam-ro, Seongbuk-gu, Seoul 02841, Republic of Korea

[b]Korean Peninsula Infrastructure Cooperation Team, Korea Institute of Civil Engineering and Building Technology (KICT), Goyang-si, Gyeonggi-do 10223, Republic of Korea

[c]Department of Internal Medicine, College of Medicine, Korea University, 148 Gurodong-ro, Guro-gu, Seoul 08308, Republic of Korea

[d]College of Pharmacy, Korea University, 2511 Sejong-ro, Jochiwon-eup, Sejong 30019, Republic of Korea

[e]Biomedical Research Center, Korea University Guro Hospital, 148 Gurodong-ro, Guro-gu, Seoul 08308, Republic of Korea

[f]KU-KIST Graduate School of Converging Science and Technology, Korea University, 145 Anam-ro, Seongbuk-gu, Seoul 02841, Republic of Korea

*Corresponding Author
Hee-Deung Park
- Address: School of Civil, Environmental and Architectural Engineering, Korea University, 145 Anam-ro, Seongbuk-gu, Seoul 02841, Republic of Korea
- Phone: +82-2-3290-4861
- FAX: +82-2-928-7656
- E-mail: heedeung@korea.ac.kr

EPS analysis

Overnight cultured PA14 strain (OD at 595 nm = 0.05) diluted in AB medium treated with either compound **30** or tobramycin was aliquoted in borosilicate bottles. After incubation at 37 °C for 24 h, the OD of the suspended cells was measured at 595 nm using a UVmini-1240 spectrophotometer (Shimadzu, Kyoto, Japan). Biofilm cells attached to the bottles were washed with PBS and scraped with 0.01 M KCl. The collected biofilm cells were disrupted using a sonicator (VCX 750, SONICS, Newtown, CT, USA) for 4 cycles of 5 s of operation and 5 s of pause at 20% amplitude. The sonicated biofilm cells were filtered through a 0.22 μm membrane filter.

For carbohydrate analysis, a mixture of 250 μL of filtrate and 750 μL of 99.9% sulfuric acid (Junsei, Tokyo, Japan) was incubated at 25 °C for 30 min. Then, 150 μL of 5% phenol was added to the mixture, and the mixture was incubated at 90 °C for 5 min. The amount of carbohydrate was quantified at OD at 490 nm and normalized at OD at 595 nm.

For protein analysis, a mixture of 200 μL of filtrate and 1,000 μL of Lowry reagent (Sigma-Aldrich) was incubated at 25 °C for 10 min. Folin-Ciocalteu reagent (100 μL; Sigma-Aldrich) was added to the mixture and incubated at 25 °C for 30 min in the dark. The amount of protein was quantified by measuring OD at 750 nm and normalizing at OD at 595 nm.

Growth inhibition test

PA14 strain (OD at 595 nm = 0.01) cultured overnight was diluted in AB medium with either compound **30-** or tobramycin treatment. On one hand, the diluted cells were incubated at 37 °C for 24 h with constant shaking (200 rpm) for planktonic cells, while on the other, biofilm cells were prepared using the PA14 strain (OD at 595 nm = 0.05), and borosilicate bottles were incubated at 37 °C for 24 h without agitation. The OD of the suspended cultures at 595 nm was measured on a VICTOR x5 multimode plate reader (PerkinElmer, Waltham, MA, USA). The suspended cultures were serially diluted with LB medium and spread on LB agar plates. After incubation at 37 °C for 24 h, the number of colonies on the agar plates was counted.

CdrA binding test

P. aeruginosa transformed with *cdrA*-*lacZ* fusion plasmid was used to evaluate c-di-GMP levels. The *cdrA* reporter strain (OD at 595 nm = 0.3) treated with chemicals (compound **30** or tobramycin) was incubated at 37 °C for 20 h. Optical density (OD) at 595 nm of the incubated reporter strain (100 μL) was measured using a spectrophotometer. The remaining reporter strain was treated with chloroform for 10 min, following which 10 μL of the suspension was used to measure luminescence using a Tropix plus kit. The luminescence value (*cdrA* expression) was normalized to that of the suspended cells (OD at 595 nm) to estimate c-di-GMP levels.

Membrane permeability test

The permeability of the outer membrane was determined using the NPN assay. The overnight cultured PA14 strain was washed and resuspended in 0.5% NaCl solution. About 150 μL of the cell suspension (OD at 595 nm = 1.0) was adjusted to pH 7.2-7.3 by adding 1.0 M phosphate buffer (pH 7.2). Compound **30** (0-100 μM), tobramycin (0-60 μM), and 3 μL of 1 mM NPN solution (Sigma-Aldrich) were added to the suspension. Fluorescence intensity (excitation wavelength: 350 nm, emission wavelength: 420 nm) was measured using a VICTOR ×5 multimode plate reader.

The permeability of the inner membrane was determined using the ONPG assay. PA14 strain cultured overnight which was washed and resuspended (OD at 595 nm = 1.2) with 0.5% NaCl solution was adjusted to pH 6.8-7.3 by adding 1.0 M phosphate buffer (pH 7.2). The suspension with compound **30** (0-100 μM), tobramycin (0-60 μM), and 15 μL of 30 mM ONPG solution (Sigma-Aldrich) was measured at an OD of 420 nm.

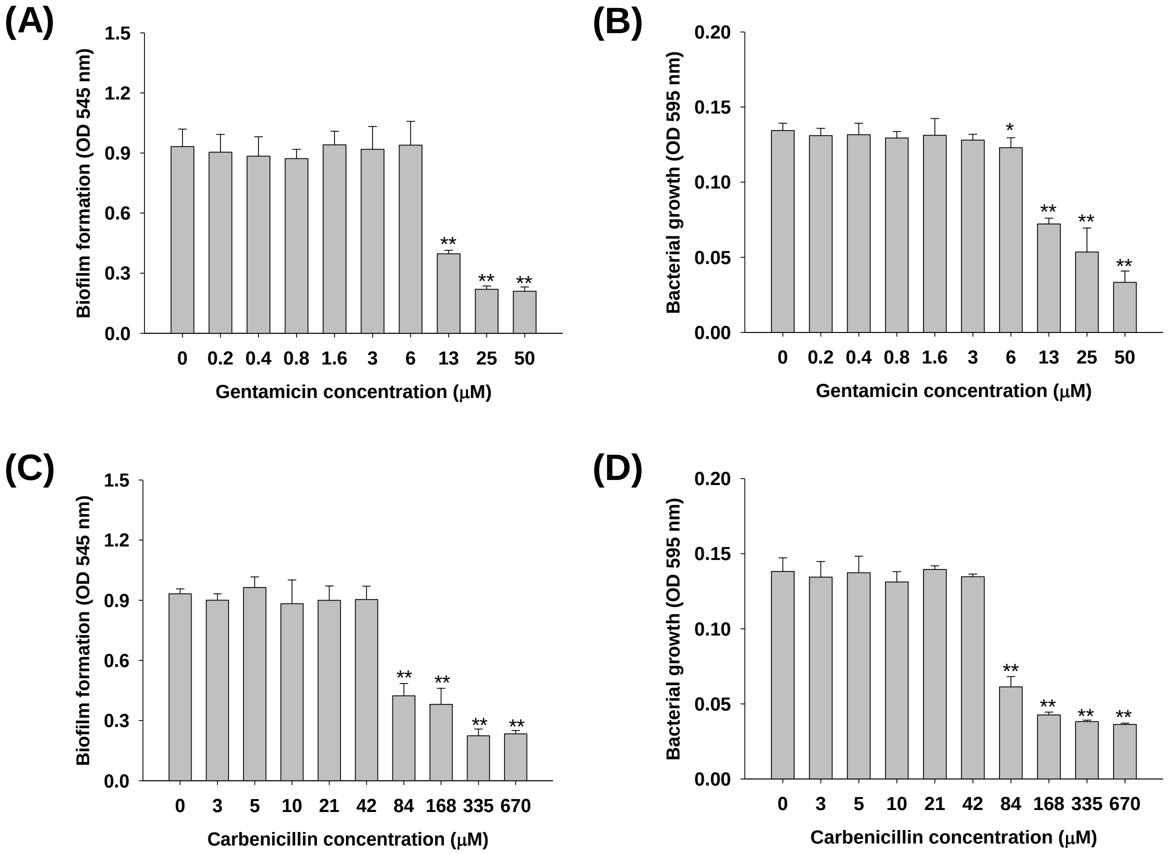

Fig. S1. Inhibition of biofilm formation and bacterial growth of *P. aeruginosa* by gentamicin (0-50 μM) or carbenicillin (0-670 μM) for 24 h in static conditions. (A) Biofilm formation by gentamicin. The biofilm formation was quantified by measuring the OD at 545 nm of the biofilm cells stained with crystal violet. (B) Bacterial growth upon gentamicin treatment. Bacterial growth was quantified by measuring the OD at 595 nm of the bacterial suspension. (C) Biofilm formation upon carbenicillin treatment. (D) Bacterial growth upon carbenicillin treatment. (**) P<0.005 and (*) P<0.05 compared with the control.

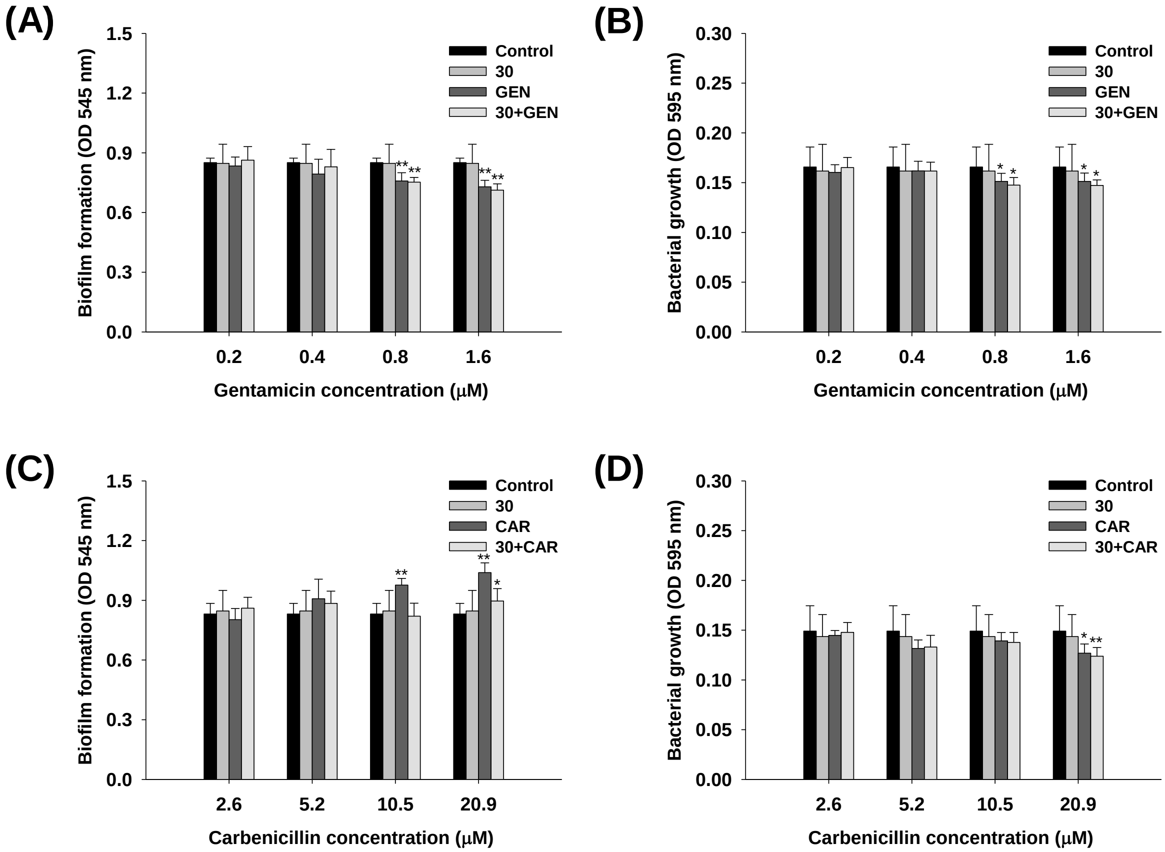

Fig. S2. Inhibition of biofilm formation and bacterial growth of *P. aeruginosa* at different combinations of compound **30** (30; 1 μM) and gentamicin (GEN; 0.2, 0.4, 0.8, and 1.6 μM) or carbenicillin (CAR; 2.6, 5.2, 10.5, and 20.9 μM) for 24 h in static conditions. (A) Biofilm formation upon treatment with the combination of compound **30** and gentamicin. (B) Bacterial growth upon treatment with the combination of compound **30** and gentamicin. (C) Biofilm formation upon treatment with the combination of compound **30** and carbenicillin. (D) Bacterial growth upon treatment with the combination of compound **30** and carbenicillin. (**) $P<0.005$ and (*) $P<0.05$ compared with the control.

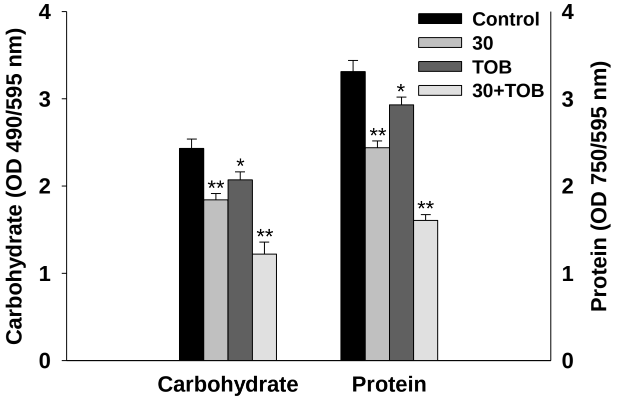

Fig. S3. The amounts of EPS components (carbohydrate and protein) in *P. aeruginosa* biofilm cells upon treatment with the optimum combination of compound **30** (30; 1 μM) and tobramycin (TOB; 0.63 μM). Biofilm was formed in borosilicate bottles for 24 h. Carbohydrates and proteins were measured using phenol-sulfuric and Lowry methods, respectively. (**) $P<0.005$ and (*) $P<0.05$ compared with the control.

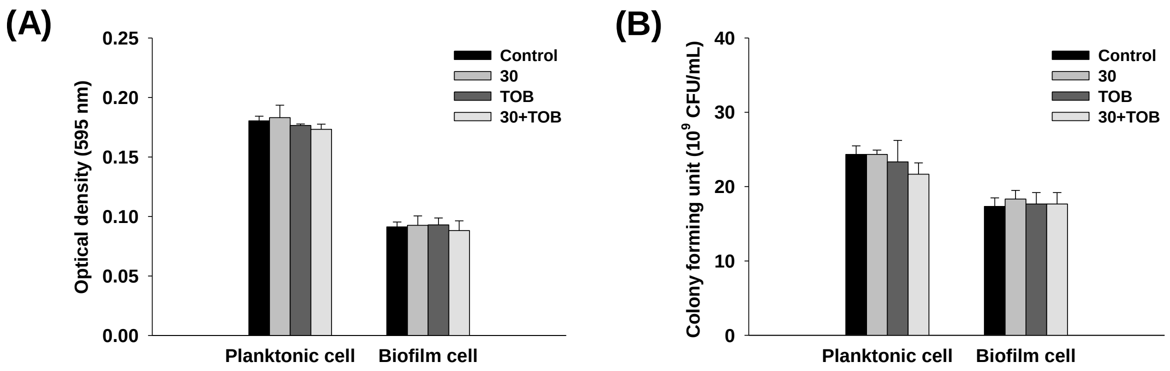

Fig. S4. Growth inhibition in planktonic and biofilm cells of *P. aeruginosa* upon treatment with the optimum combination of compound **30** (30; 1 μM) and tobramycin (TOB; 0.63 μM). While *P. aeruginosa* was incubated for 24 h with constant shaking for planktonic cells, it was incubated for 24 h without agitation in borosilicate bottles for biofilm cells. (A) Measurement of growth inhibition by evaluating the optical density of the bacterial suspension at 595 nm. (B) Measurement of growth inhibition by counting colonies of bacterial cells.

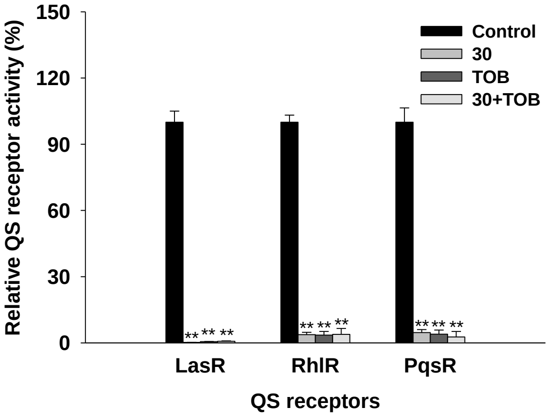

Fig. S5. Agonism activities against various QS receptors, LasR, RhlR, and PqsR, at the optimum combination of compound **30** (30; 1 μM) and tobramycin (TOB; 0.63 μM). Signal molecules (0.01 μM OdDHL for LasR, 10 μM BHL for RhlR, and 1 μM PQS for PqsR) were used as the respective positive controls. (**) P<0.005 compared with the control.

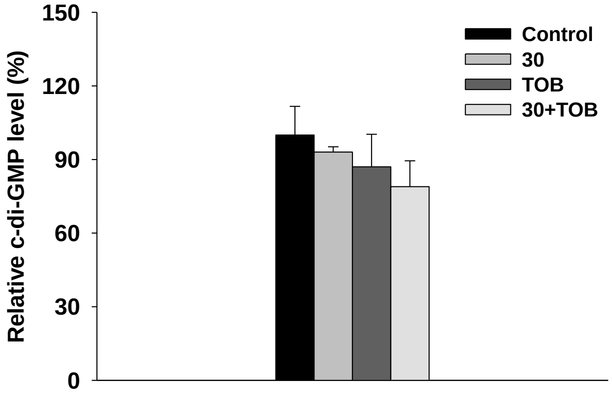

Fig. S6. The relative c-di-GMP level in *P. aeruginosa* upon treatment with the optimum combination of compound 30 (1 μM) and tobramycin (0.63 μM). c-di-GMP level was evaluated by measuring *cdrA* gene expression using specific reporter strain.

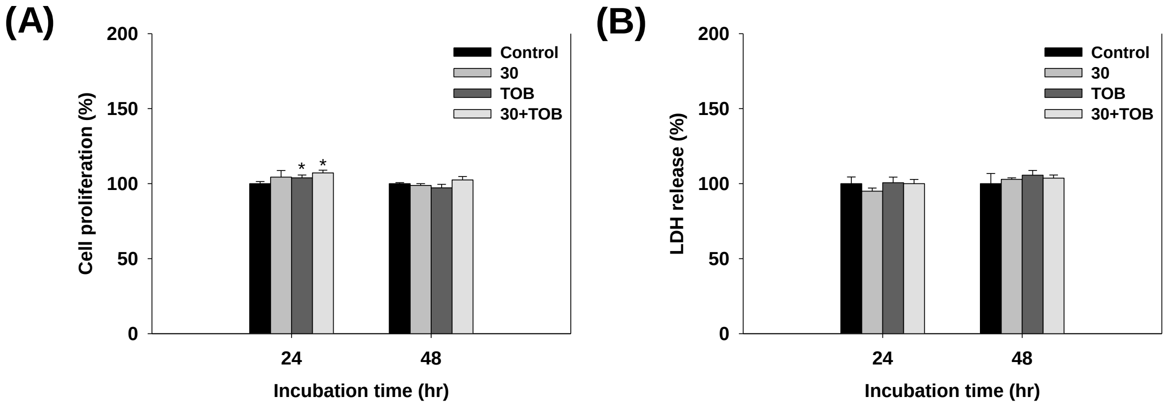

Fig. S7. Cytotoxic effects of human lung epithelial cells upon treatment with the optimum combination of compound **30** (1 μM) and tobramycin (0.63 μM) at 24 and 48 h of incubation. (A) The proliferation of lung cells upon combined treatment by MTS assay. (B) Evaluation of LDH release from lung cells upon combined treatment, by LDH assay. (*) $P<0.05$ compared with the control.

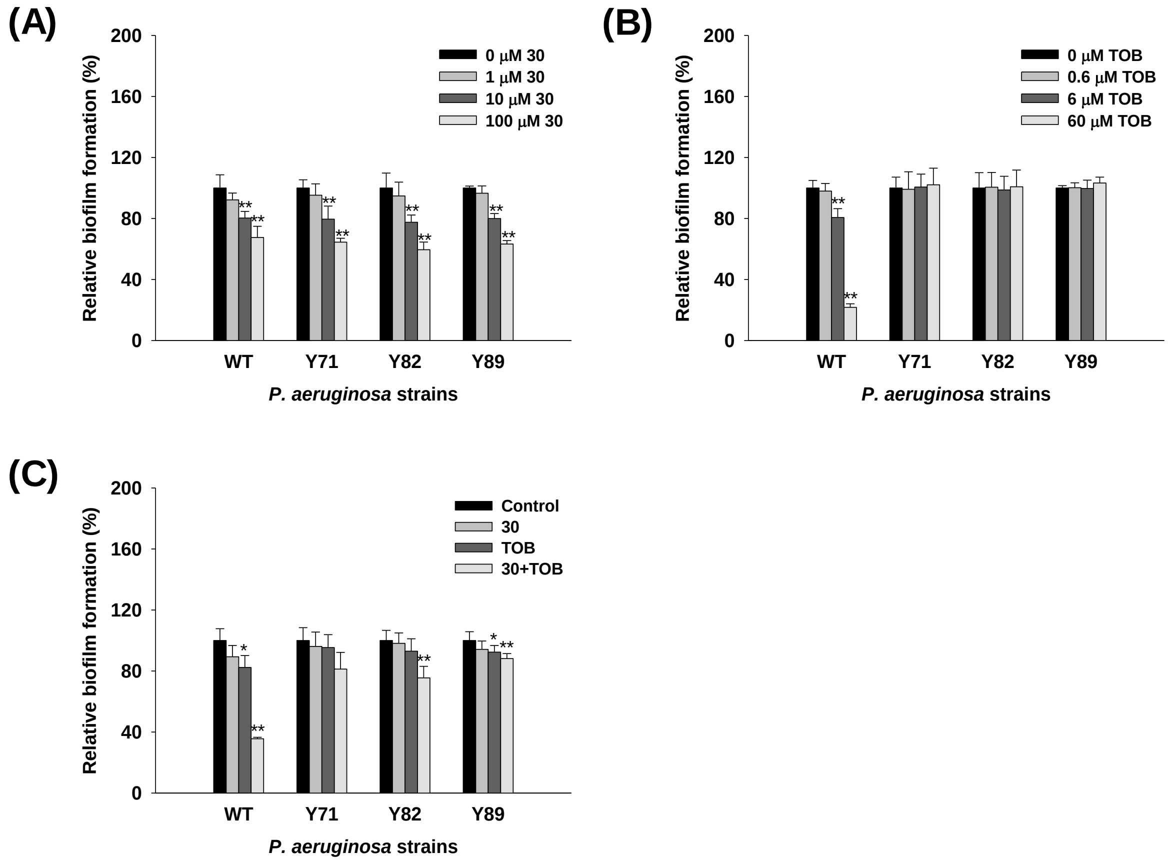

Fig. S8. Relative biofilm formation in wild-type (WT) and tobramycin-resistant *P. aeruginosa* strains (Y71, Y82, and Y89) upon compound **30** (30) or tobramycin (TOB) treatments. Biofilm was formed in static conditions for 24 h, and the color changes of stained biofilm cells were measured. (A) Biofilm formation upon compound **30** treatments (0-100 μM). (B) Biofilm formation upon tobramycin treatments (0-60 μM). (C) Biofilm formation upon treatment with the optimum combination of compound **30** (1 μM) and tobramycin (0.63 μM). (**) P<0.005 and (*) P<0.05 compared with the control.

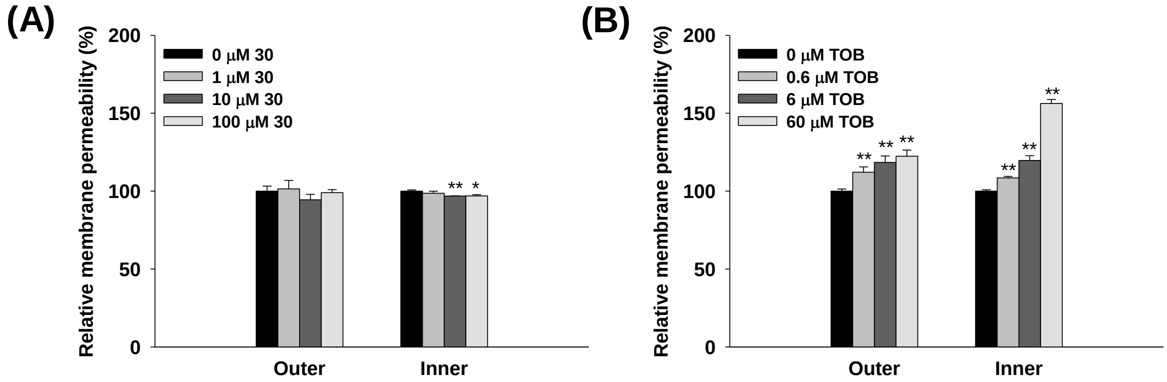

Fig. S9. Relative membrane permeability in *P. aeruginosa* by compound **30** or tobramycin treatment. The permeability of outer and inner membranes was evaluated using NPN and ONPG assays, respectively. (A) Relative membrane permeability in *P. aeruginosa* upon compound **30** treatment (0-100 μM). (B) Relative membrane permeability in *P. aeruginosa* upon tobramycin treatments (0-60 μM). (**) P<0.005 and (*) P<0.05 compared with the control.

Table S1. Minimum inhibitory concentration (MIC) and minimum biofilm eradication concentration (MBEC) of chemicals on *P. aeruginosa*.

**Chemicals**	**MBEC (μM)**	**MIC (μM)**
Compound **30**	3	> 100
Tobramycin	5	10
Gentamicin	13	50
Carbenicillin	84	168

Table S2. Adjuvant effects between compound **30** and gentamicin or carbenicillin.

No.	QS inhibitor	Antibiotics		FICI	
	**30 (µM)**	**GEN (µM)[a]**	**CAR (µM)[b]**	**Value**	**Effect[c]**
1	1	0.2		0.28	Adjuvant
2	1	0.4		0.29	Adjuvant
3	1	0.8		0.32	Adjuvant
4	1	1.6		0.38	Adjuvant
5	1		2.6	0.29	Adjuvant
6	1		5.2	0.32	Adjuvant
7	1		10.5	0.39	Adjuvant

[a]Compound **30** (3 µM) and gentamicin (13 µM) were used to calculate the FICI value. [b]Compound **30** (3 µM) and carbenicillin (84 µM) were used to calculate the FICI values. [c]FICI value < 0.5, was classified as an adjuvant effect.

Table S3. Primer sets of QS genes used in this study.

Primer name	Target gene	Sequence (5’ → 3’)	GC (%)	Tm (℃)	Product size (bp)
lasR-F	*lasR*	ACG CTC AAG TGG AAA ATT GG	45.00	60.11	247
lasR-R	*lasR*	GTA GAT GGA CGG TTC CCA GA	55.00	59.93	
lasB-F	*lasB*	AAG CCA TCA CCG AAG TCA AG	50.00	60.25	264
lasB-R	*lasB*	GTA GAC CAG TTG GGC GAT GT	55.00	60.00	
rhlR-F	*rhlR*	AGG AAT GAC GGA GGC TTT TT	45.00	60.07	231
rhlR-R	*rhlR*	CCC GTA GTT CTG CAT CTG GT	55.00	60.13	
rhlA-F	*rhlA*	CGA GGT CAA TCA CCT GGT CT	55.00	60.11	208
rhlA-R	*rhlA*	GAC GGT CTC GTT GAG CAG AT	55.00	60.42	
pqsR-F	*pqsR*	AAC CTG GAA ATC GAC CTG TG	50.00	59.97	238
pqsR-R	*pqsR*	TGA AAT CGT CGA GCA GTA CG	50.00	60.01	
phzC1-F	*phzC1*	AGC GGA TTC TCA AGG GCT AT	60.19	50.00	185
phzC1-R	*phzC1*	GTG GGT CGA ACC GAG ATA GA	60.07	55.00	
proC-F	*proC*	GGC GTA TTT CTT CCT GCT GA	60.35	50.00	236
proC-R	*proC*	CCT GCT CCA CTA GTG CTT CG	61.12	60.00	
